# ‘Essentially it's just a lot of bedrooms’: architectural design, prescribed personalisation and the construction of care homes for later life

**DOI:** 10.1111/1467-9566.12747

**Published:** 2018-04-27

**Authors:** Sarah Nettleton, Christina Buse, Daryl Martin

**Affiliations:** ^1^ Department of Sociology University of York York UK

**Keywords:** ageing, nursing home/residential care, social care, ethnography, interviewing (qualitative)

## Abstract

This article draws on ethnographic data from a UK Economic and Social Research Council (ESRC) funded study called ‘*Buildings in the Making’*. The project aims to open up the black box of architectural work to explore what happens between the commissioning of architectural projects through to the construction of buildings, and seeks to understand how ideas about care for later life are operationalised into designs. Drawing on recent scholarship on ‘materialities of care’ and ‘practising architectures’, which emphasise the salience of material objects for understanding the politics and practices of care, we focus here on ‘beds’. References to ‘beds’ were ubiquitous throughout our data, and we analyse their varied uses and imaginaries as a ‘way in’ to understanding the embedded nature of architectural work. Four themes emerged: ‘commissioning architectures and the commodification of beds’; ‘adjusting architectures and socio‐spatial inequalities of beds’; ‘prescribing architectures and person‐centred care beds’; and ‘phenomenological architectures and inhabiting beds’. We offer the concept *prescribed personalisation* to capture how practising architectures come to reconcile the multiple tensions of commodification and the codification of person centred care, in ways that might mitigate phenomenological and serendipitous qualities of life and living in care settings during later life.

## Introduction

This article explores the working practices of architects designing for social care in later life, and the development of residential care homes and extra care housing in particular. It draws on interview and observational data generated from an ethnography of architects working in the UK. The aim of the study was to explore what happens between the commissioning of architectural projects and the construction of buildings: to open up the black box of architectural work by examining practices in situ and seek to understand how ideas about care are engineered into designs. This is important because architectural design work shapes the delivery of care in ways that are overlooked by sociologists (Martin *et al*. 2015). To date, with some few notable exceptions (Gieryn 2002, Yaneva 2009) there are few empirical sociological studies that explore how buildings get commissioned and constructed and, as far as we can ascertain, virtually none in social care sector. What the extant literature on architecture more generally does indicate is that design and construction is as much social and political as it is technical and aesthetic (Till 2009).

In this article we focus on beds and bedrooms as a way in to unpack these issues, prompted by a comment from an architect who, during a discussion about approaching care home design, said ‘essentially it's just a lot of bedrooms’. This pithy comment, echoed by others, reflects a dominant trope found in both policy and design discourses across the social care sector. We argue that it is significant because a focus on ‘bedrooms’ – or the more common euphemistic reference to ‘beds’ – reflects and encodes ideas about care. It seems pertinent to ask: why are ‘beds’ foregrounded in the design and production of buildings for later life residential care? What kinds of ‘care’ and living are possible when ‘beds’ comprise spaces for dwelling in later life? Bed spaces, like other spatial forms, are not neutral but implicate social relationships and invoke subjectivities (Lefebvre 1991, Pile and Thrift 1995). Our aim in this article therefore is to unpack the salience of ‘beds’ and ‘bedrooms’ by exploring their varied uses and imaginaries throughout our data, and examine their ‘making’ and ‘remaking’ – as a way in to cast light on the embedded nature of architectural work in the context of formal care settings of later life.

We comment in more detail below on the salience of the multiple framing of ‘beds’ in relation to health and social care policies and find that the issue of care ‘beds’ taps into and reflects debates on commodification, social inequalities and the lived experiences of care in later life. First, however, we expand on our theoretical orientation and conceptual framings of architectural work; second, we review literatures that highlight the shifting policy and political salience of ‘beds’ in the context of health and social care; and third, we introduce our study methods before exploring our findings. Our attentiveness to ‘beds’ reflects the extent to which the design of ‘care homes’ and ‘extra care residential settings’ are mediated by the politics, economics and meanings of ‘care’ and of ‘home’. As these are subject to codification through guidance and relegation, we find that attempts to reconcile multiple tensions of, *inter alia*, health and safety, risk minimisation, surveillance, affordability, cost constraints and person centred design give rise to what we call ‘prescribed personalisation’. This in turn mitigates what we might see as the affective, lived, fluid and serendipitous qualities of ‘home’ environments built for formal care.

## Materialities of care and practising architectures

Conceptually we locate our analysis within recent scholarship on the materialities of care, ‘where mundane materialities act as a lens for (re)examining care practices in health and social care contexts’ (Buse *et al*. 2018: 245). Puig de la Bellacasa (2011), extending Latour's work on ‘neglected things’, argues that mundane artefacts are crucial yet overlooked in matters of care. Beds, and the spaces in which we find them, therefore offer a novel way in to understand the micro and macro politics of designing built environments because, as we will discuss, they are contested artefacts which carry differing notions of care, risk and value nested within their material forms. Moreover, the rooms in which they are emplaced are spatial configurations that need to be understood within the multiplicity of social, economic and cultural norms that shape their construction and comprehension.

We find that the positioning and repositioning of ‘beds’ is pertinent to an analysis of architecture because it engages with what Jacobs and Merriman (2011) refer to as ‘practising architectures’ – a concept they use to capture the pragmatics of design and in particular to highlight the range of actions, activities and actants of architectures as they unfold, and which presumes buildings to be ‘the effect of various doings’ (Jacobs and Merriman 2011: 212). What ‘materialities of care’ and ‘practicing architectures’ have in common, therefore, is the incorporation of artefacts and actants into analyses of the processes involved in the socio‐political production of built environments.

Taking this approach we can think of ‘beds’ as an example of what Latour (2004) calls ‘matters of concern’. By this he means that everyday things act relationally, because ‘participants are gathered in a thing to make it exist and to maintain its existence’ Latour (2004: 245). Moreover, everyday artefacts carry a moral dimension (Latour and Venn 2002), and certainly ‘beds’ prompt, shape, and reflect social norms and expectations (Crook 2008, Valtonen and Närvänen 2015). Beds are deeply embedded in the orchestration of common social practices, despite (or perhaps more precisely because of) their unassuming presence in our daily routines (Shove *et al*. 2007). Dant (2006) argues that such mundane objects are a critical, but overlooked, aspect of capitalism. He introduces the idea of ‘material capital’ to emphasise how economics are played out in the material stuff of the everyday:

The value in material objects that are incorporated into social life does not derive exclusively from their origins in production, from their meanings in consumption, from their practical use in everyday life or from the networks associated with their emergence as technical entities – it derives from all of these. (Dant 2006: 299)

This is more or less explicit in sociological and policy literatures on ‘beds’ for later life care and so we briefly review these debates as they are salient to our study findings.

## ‘Beds’ and changing contexts of care

The arrangement of ‘beds’ has altered in institutional settings, most noticeably with dormitories displaced by smaller wards and private rooms in boarding schools, hospitals and care homes. Crook (2008), citing Elias (2000), has demonstrated how this spatial shift reflects wider historical trends as reconfigurations of domestic settings from the 18th century saw beds to be increasingly sequestrated from communal living (Moroney 2016). Bedrooms became intimate spaces, as evident from the objects found in them, with wardrobes, mirrors, and hair brushes contributing to the embodied reproduction of the self (Crook 2008). But beds can never be wholly private spaces. As Tracey Emin's *My Bed* installation captures, the bed is at once intensely intimate yet subject to public scrutiny and judgement, caught in networks of wider cultural meaning (see Merck 2000). Body work by relatives and domiciliary workers often centres on the bed, blurring the private and public nature of the activities and relations that take place within it (Fairhurst 2007, Twigg 1999).

In the context of welfare institutions, ‘beds’ are central to spatio‐temporal forms of the regulation and maintenance of bodies. Armstrong (1998: 447) writes that a ‘hospital is a building of bricks and mortar, wards, kitchens, laundries, halls and corridors, but at the very core of its physical presence is the bed’. Moreover, he continues, there has been ‘a fundamental shift in the meaning of the space defined by the hospital bed’ (Armstrong 1998: 447). While early modern medicine constituted beds as therapeutic spaces, by the twentieth century beds came to be conceived of as dangerous sites with occupants at ‘risk’ through exposure to infection, sores, and the ‘not inconsiderable hazards of bed rest’ (Armstrong 1998: 451). ‘Controlling the bed state’ became a fraught issue and, in concert with the marketisation of health, beds became increasingly commodified (Green and Armstrong 1993: 337). As a corollary, Heartfield (2005: 25) argues, the ‘individual patient and clinical differences fade. Patients become beds and beds become numbers’.

If the hospital bed became a ‘hub of the therapeutic, investigative, administrative and financial network’ (Prior 1992: 68), similar constellations were evident in the social care sector. The ‘care home’ comprises a spatio‐temporal form that constitutes ‘old age’ as a distinct social category. It connotes a place for those unable to live independently and, in contrast to the hospital ideal, a place they are unlikely to leave. Despite this different temporality, there are resonances between social care and health care policies; notably that institutional beds became places of ‘last resort’ (Townsend 1962). This is a trend that continues, as contemporary policies valorise domestic settings as the optimal location of care (NHS England 2016) and, by implication, demonise institutional settings. The prioritisation of individual beds as opposed to the shared bedrooms of the twentieth century is stipulated in government standards for care homes, requiring that all new builds and new registrations are able to offer residents single bedroom accommodation (Department of Health 2006, MacKenzie 2014). Values of domesticity are privileged and, as explored in our data below, attempts are made to transpose these into formal care through attributes of ‘homely’ places that are ‘human’ in scale.

These developments illustrate how ‘beds’ are at once ‘matters of concern’ and critical ‘matters of care’ (Puig de la Bellacasa 2011). There is a multiplicity of actors, actants and activities associated with ‘beds’ as they come to act as synecdoches for the calculation of costs, efficiency and throughput; the administration and practices of care; lived experiences; and the design of ‘beds’ in terms of their form and function. An exploration of ‘practising architectures’ as they contribute to the fashioning of care home ‘beds’ therefore seems timely, if not overdue. Writing in 1962, Townsend noted how design of care homes is crucial yet ‘obscured’ by that lack of dialogue between architects and care providers, which in turn can mitigate good care. But how do architects approach their designs and engage with the other multiple stakeholders involved in the development of residential social care settings? Our study, the methods of which are outlined below, offers some insight into these issues.

## Study design and method

The present article draws on a corpus of qualitative interview and ethnographic data generated during an Economic and Social Research Council funded study called *Buildings in the Making* (2015–2018). The project aims:

to understand architects’ contribution to, and participation in, the design and delivery of social care settings for later life. It seeks to complement the now substantial literature on the evaluation of buildings in use by shifting the focus upstream to examine the social processes of design and construction.

The first stage of data collection involved 20 face‐to‐face qualitative interviews with 26 architectural professionals reflecting on previous projects. Ethnographic research generated further data where the research team worked with nine architectural practices and followed design projects that serve as case studies (CS). This enabled us to observe the day‐to‐day practices of architects and how designs evolve over time, and the complex factors that shape the design and build process. Case studies were selected to include variation in: the size and type of architectural practice (from small local firms to international firms with multiple offices); type of procurement model (traditional contract, design and build, design build finance operate); the model of care (including extra care housing, residential care homes and specialist dementia care homes), and type of client (including local authorities, private care providers, and third sector organisations).

Ethnographic and case study research facilitates a holistic, contextual understanding through incorporating multiple methods of data collection (Yin 2003), in this case, observations, documentary analysis and interviews. The researchers observed activities including design review meetings with architects, design team meetings and building site meetings with multiple stakeholders, public and user consultations, and building site visits. In total 172 hours of observation was completed. Detailed fieldnotes were used to record each observation, along with photographic images. Additional ‘ad hoc’ discussions and further audio‐recorded qualitative interviews were conducted with participants involved in the case study projects. This included nine further audio‐recorded discussions with architects as they talked though architectural plans and documents, and eight qualitative interviews with clients, developers and contractors. The team also conducted analysis of documentary sources including minutes of project meetings; planning documents (including design and access statements); plans; drawings, and design guidelines. Interviews and case studies are numbered to ensure anonymity, and names used in interview quotations and field notes are pseudonyms. The research was approved by the University of York ethics committee.

Data analysis involved close reading of transcripts and fieldnotes, noting down emergent themes, which were then regularly discussed within the research team. References to ‘beds’ are ubiquitous throughout the documentary, observational and interview data. If architects designing care homes focus on beds, then as sociologists interested in the social and material implications of their designs, this is where our analysis of how care might be re‐conceived relationally should focus too. We turn to our empirical data to explore how ‘beds’ act as matters of concern (Latour 2004) and matters of care (Puig de la Bellacasa 2011). Doing so allows us to understand the way ‘beds’ become commodities, play into wider spatial inequalities, build narratives of person‐centred care and draw upon cultural imaginaries of ‘home’ in speculations about how they will be inhabited in completed designs.

## Embedding architectures

### Commissioning architectures and the commodification of ‘beds’

Architects are clear that the starting point for the commissioning of residential care home projects rests on the number of ‘beds’. Those who have long standing relationships with (private sector) operators describe a ‘typical’ scenario:

Normally … you get a phone call on Friday afternoon about half past four and it'll say ‘Dave, I've found a site, how many beds can we get on it?’ and that'll be it. Because we are repeating clients we know what we are developing all the time and the initial feasibility, what it all comes to is: Is it financially viable? So if you've found a site and we can get a 20 bedroom unit on there, they'll work out some numbers and think: ‘so 20 bedrooms, I'll need staff for 20 people, I'll need a kitchen, office laundry and things like that and financially that model doesn't work (Interview 2)

This architect talks about industry standards in the UK, where ‘a home with between 60 and 80 beds would work on about an acre’ and explains that ‘everything is worked around the bedrooms because that's how many residents they have got to look after, so they know what their outgoings are for 60 and what their income is for 60 residents’. We find further examples of this during the ethnography, where discussion of potential new sites for development between architects, clients and developers begins with the question: ‘How many beds?’ followed with discussion of finances and timescales. Architecture, as Till (2009) puts it, ‘depends’ on all manner of contextual factors and here it depends on economics. Elaine explains architecture is not simply about ‘the design’, but is fundamentally shaped by the ‘reality’ of the ‘strategic context’ of funding and procurement processes (Architect CS8). Since the 1990s in the UK, large operators expanded their market share, such that by the end of the millennium the major for‐profit providers (those with 3 or more homes) controlled 30 per cent of the market (Johnson *et al*. 2010). Investments of private capital in building programmes are invariably substantial; for‐profit care home operators must generate profit for shareholders, and non‐profit providers must achieve financial sustainability. In our ethnographic research, discussion of the economics of beds was particularly common with private sector providers, but occurred across various models of care provision, including local authority funded projects. Developing and delivering social care is a costly business, margins can be tight and financial considerations are paramount, with the economics firmly anchored in ‘beds’.

Jacobs and Merriman's (2011) notion of practising architectures recognises the salience of multiple stakeholders, who they note include architects, builders, demolishers, cleaners and others. To their list we would also add banks and other lenders. Care home projects only evolve if loans are secured, and these in turn are linked to reliable projections of ‘active’ beds, as this architect explains:

The banks would work out that 11 per cent of the population over 75 need some sort of a care bed. It used to be 12.5 per cent and that's now come down to 11. Because obviously you've got the care in the community … [and] the dependency levels in care homes are a lot higher than what they were even three years ago.’ (Interview 20)

The associated calculations have to be precise, because ‘if you had the wrong ratio of staff to residents, that could create a huge premium on the cost of providing care to that bed’ (Interview 18). On some projects ‘monitoring surveyors’ hired by the banks as consultants oversee contracts, plans, specifications, and statutory agreements. Losing ‘beds’ is rarely an attractive option – as one developer (CS3) explains, clients cannot afford to ‘lose beds’. However, bedrooms may be adjusted on site if it is feasible to make them larger so that operators can charge premium rates from the residents, something we observed during the build of a residential care home for a private sector provider (CS6).

Further complicating these market processes is the role of local authorities, who may grant planning permission in return for affordable ‘beds’, or may fund projects to be run by care home operators so as to cover the costs of their investments. During the observation of one project team meeting (CS3), the director of a third sector care home operator explained that the local council (commissioning this particular care home development) had requested 40 affordable beds for local council residents. In order to make this financially viable he explained that they might have to balance this with higher rates for privately funded residents, and therefore ‘the more beds the greater efficiency.’ In the meeting, he goes on to explain that the council have different cost bandings for different types of residents, and require a certain number of beds for residents with low, high and medium levels of need. Heartfield (2005) points out different categories of beds intertwine with different clinical categories, which in turn have cost implications. As reported by architects in the study, developers ‘are deliberately targeting certain bands … because they're more lucrative’ (Interview 15). ‘Dependency levels’ in turn impact on staffing ratios and technical design considerations (NHS England 2016). In some ways, the care home is an architectural manifestation of ‘frailty’ which concretises the ‘social imaginary of ‘real old age’ as reliant on high levels of care’ (Higgs and Gilleard 2014: 10).

We see therefore how ‘beds’ are grouped in categories of, simultaneously, care need, funding source, and pricing. This results in an econometric logic whereby residents with lower levels of ‘dependency’ potentially subsidise those with higher levels and those who are financially better off subsidise those without means. ‘Beds’, then, come to act as markers of distinction that reproduce wider socio‐economic and embodied divisions. Through our attentiveness to the ‘materialities of care’, we can see how ostensibly neutral objects such as beds actively contribute to inequities of care.

### Adjusting architectures and socio‐spatial inequalities of beds

As we have seen, spatial adjustments such as the size or aspect of ‘beds’ can yield differing rates of return. Larger rooms, sometimes with higher specification and furnishings, can be marketed to the capture the ‘silver dollar’ (Interview 18):

If they are aiming at a group of clients that perhaps have more of a social background … well they would get less of a return. I would tend to say we need to go for minimum standard, but if they cater for more of a premium development, I would say we need to have a bedroom rather than 12 square metre have 19 square metre. And then depending on whether we are in [names a wealthy market town] or in somewhere else in the country, I would say what proportion do you want, 60/40? Once I've got all this basic information I know more or less the resulting footprint, and with that footprint I know whether there is a bronze, silver or gold standard, whether it will attract a cost per square metre’ (Interview 7)

References to ‘premium’, ‘gold’, and ‘bronze’ rooms resonate with the theme of homes as smart hotels and have a long history within discourses of care – Nye Bevan famously said that ‘any old people who would wish to go might go there in exactly the same way as many well‐to‐do people have been accustomed to go into residential hotels’ (Bevan cited in Bland 1999: 542). The enactment of the hotel model implies paying ‘guests’ have differential purchasing power, with some able to opt for ‘premium’ spaces, in contrast to Bevan's vision of a better standard of care being accessible to all older people (Pollock and Leys, 2004). One architect recounts the nature of discussions with home operators:

It gets into a whole economic thing, but that then comes back to the design: do we have several rooms that are larger than others, have a nice finish to it? Why would someone pay £1,000 a week to live in this room whilst this person over here is only paying £450 a week to live in that room? (Interview 8)

There is evidence that larger bedrooms are associated with well‐being and levels of satisfaction, with van Hoof *et al*. (2016: 46) finding that this is because residents are able to ‘bring more personal possessions and leave fewer items behind’ and have more options to shape their own lives and surroundings’. This ranking of ‘beds’ maps on to social hierarchies and ‘status syndrome’, which in turn are associated with discrete health outcomes (Marmot 2004). Neo‐liberal ideologies and spatial inequalities are woven into design plans which map on to geographical inequalities, with architects reporting how upmarket operators’ investments in the south‐east of England exacerbate a north‐south divide.

Practising architectures, Jacobs and Merriman (2011: 217) argue, also ‘implicates human mattering’, in the sense that spaces reproduce social relations and afford differential meanings (Pile and Thrift 1995). Certainly architectural adjustments to designs are not only shaped by negotiations about resources, but other considerations also come in to play, such as formalised care guidelines and the contemporary imperative towards person‐centred care.

### Prescribing architectures and person‐centred care beds

The prima facie case for person‐centred design is evident throughout formal building guidance. The Care Inspectorate (Mackenzie, 2014: 14), for example, note:

It is important to consider how each resident would like their room to be designed and decorated or to perhaps reflect what they had in their own home. In some care homes family and friends are able to help with the furnishing and decoration of the bedroom.

Good practice guidance recommends ‘homelike’ environments (Verbeek *et al*. 2009), providing ‘small group living rather than large scale institutional care’ (Smith 2013: 12). Designs must simultaneously comply with ‘legislation, regulations or standards’ such as building standards, health and safety, infection prevention and control (Mackenzie 2014:15). The *National Care Standards* report (Donnelley 2007) requires bedrooms in new homes to have en‐suite facilities, lockable space for personal belongings and space to entertain visitors in private, requiring a minimum 12.5 square metres of usable floor space, excluding en‐suite facilities and fitted units. Government publications direct designers to the good practice design guidance, most usually the Dementia Services Development Centre's (DSDC) audit tool which is used for the assessment and kite marking of ‘gold standard’ dementia sensitive design (Health Facilities Scotland and DSDC 2007).

Despite aspirations to domesticity, the thrust to achieve standardised attributes in tandem with the focus on the bedroom as the personalised space reinforces an architectural genotype (Dovey 2008). Care home design can replicate the formats of other commercial ventures such as student residences or chain hotels, with their repeat pattern of cellular spaces comprising a single door, a window, and en suite facilities (Dudham 2007). One architect talking with the researcher about the plans for a new sheltered housing development said: ‘that's the typical one bed and two bed apartments. So they are really easy because they are all just the same then all the way around the building’ (CS7). Notwithstanding the argument that such design templates connote transient living more than they do a sense of rootedness that we might associate with home, this also reflects the standardisation of design through architectural handbooks and government guidelines. This is the result of designs where ‘the standard is imposed upon each concrete instance to make the same parts interchangeable’ (Emmons and Michalache, 2013: 37). In addition, this standardisation is achieved through ‘the dimensional routinization of human activities’ (Emmons and Michalache, 2013: 39), imagining what activities take place in a space, and ‘how much and exactly what shape of space is required to enclose them’ (Emmons and Michalache, 2013: 40).

Design templates engineer care through the positioning of things; the placing of the bed in the middle of the room facilitates the body work of staff (Twigg *et al*. 2011) and accommodates paraphernalia such as nurse call points, hoists, wheel chairs, zimmer frames, electrical points, and alarm systems.

If they're going to use mobile hoists, [staff] need to actually be able to get a hoist around all three sides of a bed, and have someone there. […]. So it's enabling the staff to do their job well and efficiently, which can go down to the detail of … the wardrobe in the bedroom, it might have like a third door on it which, behind which will be linen pads, medication that people actually need, that sort of thing, so staff haven't got to walk miles to a central store and all the way back again. (Interview 8).

This chimes with Nord's (2011: 944) ethnography of a care home where she found ‘the bed itself was the space in the unit where the resident became most public through staff exposure’, in contrast to the intimate sequestered bed space of domestic settings we discussed above (Crook 2008). Care is conceived in a functional ‘care‐as‐provision’ model (Latimer 2013), where care prescribes person‐centredness as safeguarding rather than encouraging a re‐making of the material environment. Architectural design therefore involves a tension between concealing items for functional care (Bromley 2012), having hoists that will ‘disappear’ and preserving the bedroom as ‘domestic’ and ‘residential’.

The person centred focus emphasised in architectural practices is situated within a further tension in care home design between an emphasis on resident autonomy as set against concerns to minimise risks (Knight *et al*. 2010). This is something we frequently observed in architects’ discussions with care providers, who expressed concerns about residents with dementia ‘wandering’ or ‘falling’ (CS3). Beds in care settings, as discussed above, are conceived of as dangerous zones, echoing Fairhurst's analysis of UK government guidance for architects in the 1970s, where she found that older people were presumed to be ‘especially at risk and vulnerable when rising from their beds in the middle of the night’ (*Design Bulletin* 1974, in Fairhurst 2007: 102).

We might think of the attempts to foster security in architectural design as a layered aspiration, figured as an affective property of homeliness and as an operational principle of minimised risk. Together, these combine into a process of *prescribed personalisation*. By this, we mean that the injunction to be person centred means that residents are encouraged to create their own spaces, yet in ways that are not of their own choosing and are constrained by the relatively inflexible processes of codification, standardisation and evidence based guides as to what people in later life need and will want.

Architectural latitude is limited by regulations and so too will be opportunities for residents ongoing practices of ‘home‐making’ (Blunt and Dowling 2006), which can take place only in the bedroom which is constructed as being synonymous with the residents ‘home’. The researchers who revisited Townsend's (1962) classic study found that although bedrooms had become more individualised, as consequence of regulations they are now less personalised.

One of the most striking and less positive changes is revealed in a comparison of bedrooms in 1958 and 2006, when we notice that some freedoms have disappeared because of health and safety restrictions. Instead of a kettle on the hob, a sewing machine or a teapot on the table in a 1958 voluntary home, there is, in 2006, a clinical room with commode, box of tissues, white radiator and hospital style bed tray. Modern restrictions mean that freedom to live anything approaching a normal, independent life, however able the resident, has disappeared’ (Rolph *et al*. 2009: 436).

This tension between personalisation, standardisation and managing risk is also expressed in the bed itself as a material object. Despite the Care Inspectorate's (Mackenzie 2014) injunction that residents should be encouraged to decorate and furnish their own bedrooms, fire regulations often preclude residents bringing their own beds, and, as the architects explained, ‘the beds are specialist anyway’ (Interview 20). Having your ‘own bed’ is significant to narratives and meanings of ‘home’ (Hatfield, 2010), and like other material objects such as clothing, beds are shaped and moulded over time by the direct contact with the body (Valtonen 2015), in some cases creating a sense of ‘fit’, which is disrupted by the feel of an unfamiliar bed.

### Phenomenological architectures and inhabiting beds

As we note above, practising architectures, through ‘human mattering’ (Jacobs and Merriman 2011), recognise the interconnectedness of life, materiality and memory. Architects attend to the significance of anchoring a person in place and in the ‘home’. Many spoke to us about their parents’ or grandparents’ care needs, by reflecting on the poignant process of moving them into institutional residential settings, and pointed to the importance of ‘inhabiting’ and ‘being‐in’ architecture (Jacobs and Merriman 2011).

Architects also talked specifically about the bedroom as the ‘home’. Thus ‘home’ is imagined not as a house, a building, an institution, but as a bedroom. ‘You always start with the bedroom’ they tell us because this ‘is’ the resident's home.

When I'm designing [care homes], the primary focus is the bedroom for me, because obviously that's where you're going to be spending most of your time. What we find is that, whilst we provide really luxurious day spaces, usually when you go walking around they're empty, because the clients are in the rooms, so that has to be the main focus for me. But yes, the bedroom is the core, because that's your home at the end of the day (Interview 6).

Research corroborates this, for instance, Lovatt (2018) found that residents in care homes sometimes described their bedroom as ‘my flat’ or ‘my home’, and actively engaged in practices of home‐making through the display of material things, housework and ‘hosting’ visitors. Architects anticipate bedrooms will also be used for socialising: ‘they'll just do the birthday in the person's bedroom’ or when they have visitors, ‘people just use the bedroom’ and will ‘sit on the bed’ (Interview 1). This has resonance with studies that suggest that care home bedrooms can provide a personalised and private space for residents, and a space of activity rather than passivity (Barnes 2006), facilitated by engagement with their own ‘mundane objects’ (Nord 2011: 141). In the context of institutional care the bedroom is an ambiguous space: it is one's own ‘home’ and yet not a ‘home’ as we ordinarily think of.

As many studies have found, attempts to orchestrate residential care homes as authentically ‘domestic’ are insurmountable because they are fundamentally public institutions, and invariably there are limits to the degree of privacy and autonomy that can be enabled in these spaces (Buse and Twigg 2014; Hockey 1999; Reed‐Danahey 2001). While modern domestic bedrooms are back‐staged spaces for psycho‐social retreat (Crook 2008), in institutional settings repair work through the choreography of materials is undertaken to try to reassemble and reconcile the bedroom as ‘home’. Architects describe how they seek to create a sense of being in the world through facilitating the keeping of personal possessions, through what Latimer and Munro (2009: 318) would refer to as ‘giving room to things’. This is illustrated by an exchange during a public consultation meeting, which involved the architects discussing plans for a new care home with residents from the local neighbourhood:

Nick [the project architect] talks about being ‘very passionate about the bay window’ in the bedroom, which gives room for ornaments, photographs, etc. A member of the public is a bit more critical and he says ‘so you are living out of a suitcase really’, to which Nick replies that it is an adjustment as it is a ‘nursing home not a flat’, but the man says ‘but it's not much good if you are living there!’ (CS3 fieldnotes, public consultation).

While Nick emphasises the importance of space for personal things, the local resident questions the idea that the bedroom in a care home can ever really represent a ‘home’ (cf. Fairhurst 1999). Throughout this particular project, Nick is keen to retain ‘his’ bay window which is symbolic of homeliness, having already made compromises on things such as the use of aluminum window frames to meet environmental strictures, frustrating his choice of wood frames which he thinks make for a ‘warmer homely feel’ (DTM fieldnotes).

We see in our data how architects are keen to entwine design with dwelling; as one suggested, we ‘try to push the client to get a sense of life in the bedroom’ and consider what ‘the space would feel like, rather than a kind of functional list’:

most briefs essentially are sort of functional; metre square driven and strained of love or life or vitality, of human beings. And it's almost like saying we shouldn't do that, briefs actually need to be infused with a vitality. (Interview 12)

We find traces here of Heidegger's (1978) notions of ‘dwelling’ suggesting a phenomenological architecture (Pallasma 2005) and what Schillmeier and Heinlein (2009) call the ‘canniness of home’, to imply a reassurance and comfort that can accompany the familiarity of domestic space. Angus *et al*. (2005) further draw attention to the multisensory nature of place and so support the call by Bille *et al*. (2015: 37) to take more seriously ‘the co‐existence of embodied experience and material environment’. What we refer to above as ‘*prescribed personalisation*’, in the form of guidelines and assessment tools rooted in rationalist, evidence‐base discourse, rubs up against phenomenological architectures, such that some architects feel these protocols can drain spaces of life. Prescriptions on the colours of wall surfaces, materials for flooring, the positioning of doors, pictures, photographs or furniture to meet functional needs of older people (Health Facilities Scotland and DSDC 2007) can seem antithetical to ‘home‐making’ as an on‐going set of participatory practices (Blunt and Dowling 2006), which continues within the context of care.

The conundrum of making ‘beds’ that are simultaneously commercially viable, comply with the dictats of person‐centred care, and facilitate a sense of inhabitation and ‘home’ is core to practising architectures of care. Some architects, as we saw above, articulate a poetics of space ‐ ‘love’, ‘life’ and ‘vitality’ – revealing a phenomenological architecture as a foil to rationalist approaches and encoded ‘good practice.’ These accounts chime with Bachelard (2014 [1958]: 28), who writes about the house as materially and metaphorically affording opportunities for onerism or reverie:

[I]f I were asked to name the chief benefit of the house, I should say: the house shelters daydreaming, the house protects the dreamer, the house allows one to dream in peace. […] Therefore, the places in which we experienced daydreaming reconstitute themselves in a new daydream, and it is because our memories of former dwelling‐places are relived as daydreams that these dwelling places of the past remain in us for all time.

Bachelard elegantly suggests that, through their distinct spatial forms and imaginaries, cottages, grand country mansions and suburban bungalows possess different virtues, dreams and aspirations. One architect evokes similar domestic imaginaries, crafting the bedroom as synonymous with the house which is quintessentially home.

The reality is with this particular building, this is not somewhere people are temporarily, this is where they are till the end of their life, so I was quite keen that we wouldn't do what I saw everywhere else which was four walls, a window and a door, that was it. So we had this idea of expressing each room as like a little house, which in a way is giving it its own roof, but I had a very practical idea where sunlight, is supposed to be a great stimulant to dementia patients, when we have sunlight, and obviously with that plan you've got rooms facing in all sorts of different directions, so if you have a section like I've got there, you can guarantee that you're always going to get sunlight into the room one way or the other, because it's coming from two different sides. So that's what that was all about, and then on top of that we took a window and generally turned them into little corner window seats. In fact the original idea was that was going to be big enough for a relative to stay the night, but it got value engineered down a bit, so it still became a corner window seat. (Interview 10)

A lengthy quotation, yet still a succinct and emblematic articulation of the aesthetic aspirations, professional knowledges and practical tensions with and within which architects work. Thus, we can trace in these words an interlocking attention to the environmental, material and social factors that influence design: in this case, the incorporation of the natural light of the diurnal rhythm and windows providing views, and a roof of one's own, creating opportunities for hospitality to guests. However, this sensitivity to the physicality and sociality of architecture is also embedded in commercial contexts, as the reference to the process of ‘value engineering’ confirms.

## Discussion

A point of departure for this article was a comment by one architect who said of the care home: ‘Essentially it's just a lot of bedrooms, but you can manipulate it and try to disguise it or improve it all the time (CS6), a view voiced by other architects throughout our study. Such strategies are integral to ‘practising architectures’, and take place with the wider economic, political, and regulatory contexts on which architecture ‘depends’ (Till 2009). Our analysis of the various uses, meanings, imaginaries and networks that, to use Latour's (2004) word, ‘gather’ around ‘beds’ in care home architectural projects shows how ideas and ideologies of care come to be inscribed in designs and buildings for later life. ‘Beds’, we find, are made and re‐made through a series of tussles between commercial imperatives, material considerations and professional values throughout the design process, and in a politico‐economic climate where market imperatives can exacerbate social and spatial inequalities of care provision. ‘Beds’, as a source of ‘material capital’ (Dant 2006), serve as a prism, revealing the ways in which political, economic and moral issues constrain designs, a finding consistent with evidence that processes of marketisation and associated rationalisation of services impacts on quality and delivery of care (Lewis and West 2014). Burstow *et al*. (2014: 189) are critical of this privileging of ‘beds’ as the site for care, arguing that: ‘No commissioner should look to commission a “bed” or a “room” – but a package of support based on outcomes each person wants to achieve’.

Interwoven with the marketisation of care is the ideology of choice and autonomy articulated in terms of person‐centred care. The imperative to ensure buildings will enable care to be centred on the individual's need to feel ‘at home’ is explicit in good practice guidance which stipulates bedrooms should have homely furnishings, en suite facilities and space for personal possessions. Care homes must facilitate residents’ autonomy, such that they will be able to make their bedroom a place of their own (Mackenzie 2014). However, as the injunction to be person centred becomes codified this can mean that residents may find themselves in domestic ‘homely’ spaces that are not of their own choosing, as they are standardised to meet the strictures of what counts as ‘dementia friendly’, safe, secure or appropriate for older bodies. We call this ‘*prescribed personalisation*’, which manifests as a series of tensions between ‘care‐as‐provision’ (Latimer 2013: 37) and a more phenomenological architecture that seeks to facilitate a different, more serendipitous sense of inhabitation, dwelling and ‘home’. As ‘beds’ are assembled and re‐assembled through a series of tussles between commercial imperatives, material considerations and regulatory demands, the potential for what Schillmeier and Heinlein (2009) call the ‘canniness of home’ is bleached out, as designers come to rely on templates for ensuring spaces meet prescribed requirements in terms of, for instance, ‘homely’ yet ‘safe’ materials, security gadgets, the positioning of the bed, and even the bed itself (cf. Rolph *et al*. 2009). Notions of care, risk, and value ‘gather’ around ‘beds’ both in their material form and in the spaces in which they are emplaced, and within the production of care homes they are ‘matters of concern’ (Latour 2004) and critical, we argue, as ‘matters of care’ (Puig de la Bellacasa 2011).

## Conclusion

With an empirical focus on the work of architects, this article has explored the design of ‘beds’ in the context of the residential care settings. We have seen how, in context of later life care policy, ‘beds’ are viewed as sites of last resort, connote notions of dependency, and are the basis of financial planning. We have also seen how ideas about ‘beds’ in care settings have shifted in concert with wider cultural notions of bed spaces; collective dormitories gave way to individual bedrooms in order to ensure privacy and respect for the intimate pscyho‐social reproduction of the body. This is in the context of approaches to design that privilege domestic over institutional models of care, which emphasise autonomy and scope for personal expression. Yet in domestic homes the bedroom is, as we have seen, a sequestrated space devoted more to the reproduction of an embodied self rather than an arena for convivial living and for the presentation of the social identity. And so while the personalisation of bed spaces in care home settings is surely welcomed, it is nevertheless prescribed and circumscribed. Occupants are encouraged to display their own personal effects on for example, a spacious bay window ledge, or place family photographs on a bedside table, or hang their own pictures, and bedrooms must be sufficiently spacious to enable residents to host relatives and friends. Personalisation in the care home therefore is prescribed and limited to bed spaces where personal choices are negotiated in the context of guidelines that dictate layout, lighting, colour and so on. But, perhaps more significantly, personalisation is also circumscribed to the bedroom itself, because the bed space appears to become synonymous with the residents’ ‘home’. This in turn prompts questions about contemporary approaches to designing for care which might want to consider care homes not so much in terms of a lot of bedrooms but more as spaces and places for living.
